# Getting Developmental Science Back Into Schools: Can What We Know About Self-Regulation Help Change How We Think About “No Excuses”?

**DOI:** 10.3389/fpsyg.2019.01885

**Published:** 2019-08-21

**Authors:** Rebecca Bailey, Emily A. Meland, Gretchen Brion-Meisels, Stephanie M. Jones

**Affiliations:** Prevention Science and Practice, Harvard Graduate School of Education, Harvard University, Cambridge, MA, United States

**Keywords:** social and emotional skills, policy, research, self-regulation, developmental science, “no excuses” schools, classroom management, school discipline

## Abstract

Research from education, psychology, and human development indicates that social and emotional skills are essential to success in school, work, and life, and that high-quality social and emotional learning (SEL) programs can benefit students’ mental health, academic achievement, and behavioral outcomes. While many schools are adopting an SEL approach, there remains a concerning gap between SEL research and policies and practices related to discipline and behavior management. Following the No Child Left Behind Act and education reform driven by a culture of high-stakes standardized testing and accountability benchmarks, there has been an increase in elementary schools adopting a “no excuses” model of education. This model is characterized by extended time in school, highly structured in-service teacher training, frequent assessments, and “zero tolerance” policies to strictly manage and control children’s behavior. These behavior policies are problematic as they run counter to what research tells us about children’s social and emotional development. Reactive and exclusionary discipline policies inhibit children’s abilities to build and practice self-regulation skills and jeopardize the relationships between students and teachers. The developmental science perspective on children’s regulatory skills suggests that the early years of school are a central context for developing and practicing self-regulation with the support of educators and peers. Research also indicates that warm, caring, reciprocal relationships based on trust are critical to learning and development. Yet, this research base is often overshadowed by pressures to improve standardized achievement scores or misinterpreted in the form of hyper-vigilance about children’s behavior in the classroom. Finally, the “no excuses” approach to behavior management is used disproportionally in schools serving low-income students of color and thus may contribute to unequal rates of suspensions and expulsions, both of which are linked to negative developmental outcomes later in life. This is particularly true for students who have experienced trauma, in part because the act of social exclusion is often re-traumatizing. This article summarizes research on self-regulation, trauma, and developmental relationships, highlights potential consequences of “no excuses” policies and practices in schools, and presents an alternative view of learning environments which promote effective self-regulation skills in young children.

Research from the fields of education, psychology, and human development point to the growing consensus that social and emotional skills are essential to success in school, work, and life (Moffitt et al., 2011; Jones et al., 2015; Jones and Kahn, 2017; Aspen Institute National Commission on Social, Emotional, and Academic Development, 2019). A number of recent studies demonstrate that high-quality social and emotional learning (SEL) programs can benefit students’ mental health, academic achievement, behavior, and life outcomes (Durlak et al., 2011; Sklad et al., 2012; Jones and Kahn, 2017; Mahoney et al., 2018). The imperative for SEL in schools grows out of a long line of similar calls for school readiness (Kagan, 1990), character education (Lickona, 1996), and educating the whole child (Noddings, 2005). Although many schools are now adopting an SEL approach in their work, there remains a concerning gap between what we know about SEL through research and policies and practices in schools related to discipline and behavior management.

Following the No Child Left Behind (NCLB) Act and education reform driven in part by a culture of high-stakes standardized testing and accountability benchmarks, there has been an increase in elementary schools adopting a “no excuses” model of education (Kahlenberg and Potter, 2014; Sondel, 2016; Lamboy and Lu, 2017; Torres and Golann, 2018). This model is characterized by extended time in school, highly structured in-service teacher training programs, frequent “high-stakes” assessments, and “zero tolerance” policies to address and manage children’s behavior (Carter, 2000; Thernstrom and Thernstrom, 2003). Common features of “no excuses” behavior policies include: (1) extremely high expectations for young children’s behavior, for example calling for 100% of students on task 100% of the time (Lemov, 2015), (2) use of negative consequences or punishment, including removal from classroom, loss of privileges, and public reprimands for minor infractions such as classroom interruptions or not correctly standing in line (Lamboy and Lu, 2017), and (3) a reliance on highly structured, adult-control of behavior, such as physically re-situating children’s hands on their desks, or rigidly prescribing in detail how children should look when they are appropriately engaged (Goodman, 2013; Lemov, 2015; Taylor, 2015). Some argue that this approach to school discipline mirrors the broken windows theory of policing (Kelling and Wilson, 1982), in which teachers are instructed to “sweat the small stuff” (Whitman, 2008) and students are given automatic consequences for behaviors like eye-rolling or squirming in their seats (Balogh, 2016; Balonon-Rosen, 2016; Golann and Torres, 2018). Often conceptualized in the context of charter management organizations (CMOs), elements of “no excuses” and exclusionary disciplinary practices can also be found in public and private schools across the United States.

The term “no excuses” grows out of a viewpoint that poverty is not and cannot be used an excuse for failing schools (Carter, 2000). In this environment, children and teachers are asked to work harder and longer, with the mindset that every minute not engaged in academic instruction is time wasted (Goodman, 2013). Success and achievement are measured through student growth on frequent assessments, and indeed natural experiments (i.e., lottery-based studies) have found that urban charter schools subscribing to the “no excuses” model have shown positive and statistically significant impacts on standardized test scores (Angrist et al., 2013; Cohodes, 2018). A recent meta-analysis estimates that “no excuses” charter schools increase student math and literacy achievement by 0.25 and 0.17 standard deviations, respectively, compared to students in traditional public schools, for each year of attendance (Cheng et al., 2017). Such findings are likely responsible in part for the expansion of “no excuses” schools through millions of dollars in corporate and federal grants, though there is no evidence to suggest that it is the disciplinary policies and practices of these schools that lead to increases in academic achievement (Dobbie and Fryer, 2013; Torres and Golann, 2018).

Unfortunately, the time pressure and anxiety caused by such accountability systems often result in the removal of topics and subjects from the school day that are not directly assessed, such as social studies and writing, and an increase in teacher-centered instruction at the expense of student-centered and cooperative, project-based learning activities that have been shown to increase student’ self-regulation skills (Au, 2007; DiDonato, 2013). We would extend this list to include even more marginalized subjects, such as social and emotional programs, music, art, gym, recess and extra-curricular courses. At the same time, in order to do well both on and beyond tests of academic progress, students must exhibit a type of focus, persistence, and self-control that requires fundamental self-regulation as well as other core social and emotional skills (Mischel et al., 1989; Blair, 2002; Howse et al., 2003; Graziano et al., 2007; McClelland et al., 2007; Morrison et al., 2010; Best et al., 2011).

This catch-22 leads many districts to resort to what appear to be quick-fix solutions relying on adult-regulation and strict control of children’s everyday behaviors. Scientific studies about the importance of grit, self-discipline, and similar skills are sometimes used to justify the “no excuses” approach to learning and behavior (Whitman, 2008; Sondel, 2016), but this approach to behavior management is a misinterpretation of research on self-regulation^1^ and paradoxically can undermine children’s opportunities to develop critical social and emotional skills (Golann, 2015; Lewis, 2015; Engber, 2016; Sondel, 2016; West et al., 2016; Golann and Torres, 2018; Soutter, 2019) – skills that are a foundation for success in school and life-long health (Jones and Bouffard, 2012). Indeed, more and more “no excuses” schools explicitly state that they focus on character, values, and other SEL-related skills. Yet in practice, the development of these mindsets, skills, and competencies are de-prioritized and undermined through school policies and practices that contradict these goals, for example by limiting students’ opportunities to practice autonomous decision making (Dishon and Goodman, 2017) and by compromising other aspects of learning and development such as the cultivation of self-determination, intrinsic motivation, and self-respect, as well as agency, identity, and civic voice (Lamboy and Lu, 2017). In addition, reactive and exclusionary discipline policies can inhibit children’s abilities to practice self-regulation skills and jeopardize the relationships between students and teachers (Ben-Porath, 2013; Barker et al., 2014; Golann, 2015).

Thus, it is important to understand which practices of “no excuses” schools are linked to academic achievement and which may undermine the very outcomes they hope to produce. Practices such as data-driven instruction, extended instructional time, high-dosage tutoring, rigorous and intensive professional development for teachers, and high expectations seem to better explain academic achievement in these schools (Dobbie and Fryer, 2013; Torres and Golann, 2018). By contrast, Golann and Torres (2018) in their review of the literature on “no excuses” disciplinary practices found that there is no evidence to suggest that “no excuses” or “zero tolerance” discipline practices are positively linked to student achievement and may in fact undermine other aspects of student success and well-being. We argue that “no excuses” practices may be actively harmful to the development of healthy social-emotional skills and behavior.

In May 2015, students filed an unprecedented class action lawsuit against the Compton Unified School District, arguing that there are dangerous and discriminatory effects of expulsion and other punitive practices, in particular for trauma- and violence-exposed children (Turner, 2015). This lawsuit reflects a pattern of particular concern: the trend of “no excuses” behavior management disproportionally impacting low-income children of color (Kahlenberg and Potter, 2014; Lamboy and Lu, 2017) and placing the burden and the blame on children when they struggle, rather than on the systems and structures that perpetuate inequities and a class-based skills gap (Lack, 2009; Sondel, 2016). A similar civil rights complaint was filed in New Orleans in 2014 against “no excuses” schools for their disciplinary practices; the complaint described suspension rates that were almost seven times greater than the state average and “a culture of hyper-discipline that is punitive and demeaning to students” (Calhoun et al., 2014, p. 4). Former teachers have described the “no excuses” learning environment as one that is militaristic, prizes silence, and strict authoritarian obedience and that seeks to rigorously control student’s bodies, including by discouraging students from health-promotive behaviors such as taking bathroom breaks and getting water (Lack, 2009; Calhoun et al., 2014; Smith, 2015).

There is clear documentation that young children of color are more likely to be sent out, suspended, and expelled from classrooms and schools for minor infractions and typical behavioral challenges (US Department of Education Office of Civil Rights, 2014). This is equally the case for other marginalized groups such as children with disabilities, including but not limited to students with attention-deficit and attention-deficit/hyperactivity disorders (ADD/ADHD), autism spectrum disorder (ASD), and emotional and behavioral disorders (EBD) (Martin, 2014; Denice et al., 2015; Losen et al., 2016). Zero tolerance and other reactionary disciplinary policies may contribute to these unequal rates of suspensions and expulsions, both of which are linked to negative developmental outcomes later in life (Meek, 2014; Skiba et al., 2014; Gregory and Fergus, 2017). This can be particularly harmful for students who have experienced trauma, in part because the act of social exclusion is often re-traumatizing (Marcus, 2014; Balogh, 2016). Finally, these policies may contribute to the exclusion of children with disabilities from “no excuses” schools altogether, when schools counsel out students who are deemed a poor fit for the school when they struggle to be successful in the “no excuses” environment (Dudley-Marling and Baker, 2012; Noguera, 2014).

The developmental science perspective on children’s regulatory skills suggests that the early years of school are a central context for developing and practicing self-regulation with the support of caregivers and peers (National Research Council and Institute of Medicine, 2000; Morrison et al., 2010; Center on the Developing Child at Harvard University, 2011). Research also demonstrates that warm, caring, reciprocal relationships based on trust are critical to learning and development (Li and Julian, 2012; Jones et al., 2013; Osher et al., 2018). To the extent that “no excuses” disciplinary approaches weaken the quality of relationships in the classroom, use shame and fear in an attempt to change behavior, limit autonomy and skill-building opportunities, damage the development of positive self-concept and self-efficacy, and disproportionately punish low-income children of color and students with disabilities, we argue they are likely to be actively harmful to children (Ryan and Grolnick, 1986; Jones et al., 2016; Lamboy and Lu, 2017). This article brings to the forefront research about safe learning environments where young children can develop and practice effective self-regulation-related skills and argues that all children deserve equal access to such educational settings.

## What we know: Self-Regulation Development

### Self-Regulation Skills Develop and Become Increasingly Sophisticated Over Time

Children are not born able to manage their behavior in socially appropriate ways: to focus and persist despite frustration, to remember and follow directions, or to communicate effectively through conflict (National Research Council and Institute of Medicine, 2000). Science tells us the area of the brain responsible for focus, memory, and self-control is just beginning to mature during the preschool and early school years, and as a result, school-age children undergo substantial growth in their abilities to manage emotions, behavior, and attention (Kopp, 1982; Garon et al., 2008; Calkins and Marcovitch, 2010; Center on the Developing Child at Harvard University, 2011; Diamond and Lee, 2011). Consistent with this view, teachers report that students arrive at school with limited skills in the area of managing their own behavior. In a national survey, over half of the teachers report that children enter kindergarten with specific challenges and the most common challenge (46%) is difficulty following directions (Rimm-Kaufman et al., 2000). This makes sense from a developmental perspective. Regulation-related skills are learned over time as children develop and are supported by specific changes in the brain, especially in the pre-frontal cortex and anterior cingulate cortex regions (Diamond and Taylor, 1996; Rueda et al., 2011; Munakata et al., 2012). Self-regulation is also learned over time through experiences in the environment, most significantly through interactions with caregivers and peers (Shonkoff et al., 2000; Calkins and Leerkes, 2011).

The earliest signs of regulation appear during the toddler and early childhood years, when children are first able to choose a non-preferred response through conscious control (Kopp, 1982; Bronson, 2000; Kochanska et al., 2000; Calkins, 2007). As the child develops, this nascent version of regulation goes through a process of differentiation, by which simple skills become more specific and targeted to a range of situations (Bailey and Jones, 2019). Over time, these skills become even more differentiated, which can be reorganized and integrated, thereby leading to more complex skills and behaviors (Werner, 1957). Between the ages of 4 and 7 years old, children establish foundational regulation-related skills, or what might be described as the “core regulatory processes” of inhibitory control, attention control, set shifting, and working memory (Bailey and Jones, 2019). Each new set of challenges a child encounters requires learning how to use these skills in distinct ways: self-regulation in a math class looks different than self-regulation in gym, on the playground, or in one’s living room. A child may be successful at focusing and managing behavior in one setting, while seeming out of control in another, and it is normal for children to regress when they are hungry, tired, or during times of transition and new routines. While researchers consider the early school years to be a particularly important time to help children practice and build their self-regulation skills, individuals continue to build and expand these skills well into adulthood, reflecting the protracted development of the pre-frontal cortex and the evolving environmental demands of adolescence and adulthood (Best and Miller, 2010; Center on the Developing Child at Harvard University, 2011; Bailey and Jones, 2019). Indeed, adolescence is an important time of additional brain growth and reorganization of the prefrontal cortex and related executive function, self-regulation, and social skills (Diamond, 2002; Paus, 2005; Blakemore and Choudhury, 2006; Crone, 2009). This developmental trajectory suggests the need to continue to provide support for the growth of self-regulation-related skills throughout the school-age years.

Furthermore, a person’s ability to use self-regulatory skills in a given context is sensitive to both local demands and available support (Sameroff, 2010). Even for adults who have had years of experience, new contexts often require an adjustment period to translate and practice old skills. Demanding that children be able to draw upon and consistently deploy newly emerging skills, regardless of context, is simply not realistic. Punishing children for these failures often interferes with motivation, self-efficacy, and engagement in school (Ryan and Grolnick, 1986), which may lead to additional negative consequences. Failure to recognize that the early school years are a time when children need adult support in this area of development will cause educators and others who work with children to miss out on these critical periods for teaching and learning important self-regulation and self-management skills.

### Self-Regulation Develops in the Context of Relationships and Interactions

Transactional and relational system theories emphasize that development takes place not linearly in a vacuum, but through complex interactions between children and their environments (Lerner, 1978; Vygotsky, 1978; Bronfenbrenner, 1979; Sameroff, 2009, 2010; Osher et al., 2018). Proximal factors such as parenting, teaching, and adults’ own self-regulation skills are critical influences on regulation-related development, and thus may be the most impactful targets of change in intervention efforts (Sameroff and Fiese, 2000; Sameroff, 2009, 2010; Bailey and Jones, 2019). These relationships and everyday interactions are seen as a crucial “active ingredient” of optimal human development (National Scientific Council on the Developing Child, 2004). For example, warm, positive relationships, and low levels of conflict with kindergarten teachers are associated with children’s academic achievement and positive feelings about school, and conversely, student-teacher relationships characterized by conflict and dependency in kindergarten are associated with long-term academic and behavioral challenges (Birch and Ladd, 1997; Hamre and Pianta, 2001; Pianta and Stuhlman, 2004; O’Connor and McCartney, 2007; Rudasill, 2011). In order to promote positive development and transfer of skills, teaching relationships should be characterized by emotional attachment, reciprocity, a balance of power that gradually shifts toward the child, and scaffolded for progressive complexity (Vygotsky, 1978; Li and Julian, 2012). These scaffolds and supports should be responsive to the child’s developmental stage and context, providing emotional security while simultaneously promoting skill building (Osher et al., 2018; Morawska et al., 2019).

Consistent with this view of scaffolding and gradual release of adult control, self-determination and person-environment fit theories highlight the importance of adults who foster relatedness, competence, and autonomy in the developing child and who gradually minimize their levels of control as children’s desire and capacity for autonomy increases (Eccles et al., 1991; Gagné and Deci, 2005). They summarize that “the optimal level of classroom structure and control would satisfy two conditions: (1) it would mesh well with the student’s current level of maturity and need for both control and autonomy, and (2) it would pull the students along a developmental path toward higher levels of maturity and independence” (Eccles et al., 1991, p. 55). In a study of 140 elementary children, Ryan and Grolnick (1986) found that children who perceived their classroom environment to be a place where they had more autonomy also reported higher levels of self-esteem, perceived academic competence, mastery motivation, and sense of internal control over outcomes. Conversely, children in “controlling” classrooms where they perceived “powerful others” to be in charge reported lower levels of intrinsic motivation, self-esteem, and perceived competence. Children with autonomy-oriented teachers were also more likely to write stories in which the child protagonist was an “origin character,” meaning that they were depicted as responsible, important, and having an internal locus of causality. These stories also depicted less aggression and violence than those of children whose teachers were characterized as controlling (Ryan and Grolnick, 1986).

Taken together, this body of work suggests the central role of promoting effective self-regulation and social and emotional development as a foundation for learning. Reciprocal relationships that are warm, responsive, and foster a sense of autonomy through developmentally appropriate scaffolding and gradual release of control show the most promise for both immediate and long-term positive developmental outcomes.

### Self-Regulation Development Can Be Disrupted by Stress and Trauma

Finally, there is evidence that stress and trauma can make it increasingly difficult to use self-regulatory skills (National Scientific Council on the Developing Child, 2010). Research documenting the effects of both chronic and acute stress has demonstrated that the presence of environmental stressors can limit or interfere with key executive function skills like impulse control, planning, goal-setting, and decision-making, as well as basic learning and memory (Arnsten, 1998; Raver, 2004; Noble et al., 2005, 2007; Arnsten et al., 2012). This is partially due to heightened basal levels of the hormone cortisol, associated with exposure to poverty, stress, and trauma, which impedes the functioning of the pre-frontal cortex (Lupien et al., 2001; Kishiyama et al., 2008). Children with developmental delays due to factors such as preterm birth or postnatal malnutrition, which may go unrecognized, can also exhibit challenges with self-regulation and characteristics of attention-deficit disorders (Galler et al., 1983; van Baar et al., 2009; Jaekel et al., 2016). This may look like aggression, lack of focus, forgetfulness, or disengagement – all of which are identified within the “no excuses” model as behaviors warranting punishment or public reprimand.

The humiliation of students in front of their peers through punitive and exclusionary discipline practices is not only an additional environmental stressor but may be traumatizing in and of itself (Cameron and Sheppard, 2006). Punishing children for typical behavioral challenges is counter-productive; punishing children for behavioral challenges that are exacerbated under conditions of stress or developmental delay may be actively harmful, especially if it interferes with the healthy expression and processing of grief, sadness, anger, frustration, or other emotions associated with adverse life experiences (Lamboy and Lu, 2017). Efforts to repress emotions related to trauma can lead to classroom outbursts, as well as problems down the line with rage, substance abuse, and self-harm (Cantor, 2015). Many children growing up in poverty experience periodic or chronic stressors such as hunger, homelessness, loss of a loved one, and exposure to violence (Evans and English, 2002; Cantor, 2015). The accumulation of both physical and psychosocial stressors leads to over-taxing or overwhelming the brain’s physiological stress response system which is designed to handle less-frequent, acute stressors (McEwen and Gianaros, 2010). Over time, this leads to “wear and tear” and disruptions in the brain and body’s ability to cope with external demands (Seeman et al., 2010). These experiences can put children at risk for poorer social emotional and self-regulation skills and can create the conditions for aggressive behavior and trouble establishing and maintaining positive relationships with others (Evans and Kim, 2013). Fortunately, research indicates that high-quality relationships and targeted supports during the early school years can be a buffer against the effects of trauma, improve children’s skills, and set them up for success (Jones et al., 2013; Blair and Raver, 2014; Osher et al., 2018) and that young people with higher levels of regulation-related skills are more likely to show resilience, even when exposed to toxic levels of stress (Buckner et al., 2003). This research points to an increased need within high-poverty or trauma-exposed communities for warm, nurturing, and responsive relationships with adults, in contrast to the punitive, controlling, and institutionalized responses described at some “no excuses” schools (Ben-Porath, 2013).

## What we do: The Gap Between Science and Practice

While many schools that embrace a “no excuses” approach to behavior management cite research linking non-academic skills to long-term positive outcomes (e.g., Moffitt et al., 2011; Tough, 2013; Mischel, 2014; Jones et al., 2015), there are several substantial ways that a “no excuses” approach can undermine the development of self-regulation, which is a central developmental skill and is itself linked to long-term well-being, academic achievement, and social competence (Bailey and Jones, 2019).

### “No Excuses” Expectations Are Inconsistent With Research on Child Development

“No excuses” schools emphasize high expectations for academic achievement as well as for non-academic skills such as self-control and attention (Carter, 2000; Thernstrom and Thernstrom, 2003). While an approach to schooling that emphasizes non-academic skills and social and emotional learning is supported by the literature, simply demanding high performance is different than teaching specific social and emotional skills. High expectations may have a positive impact on children (Dobbie and Fryer, 2013; Cohodes, 2018), but if high expectations are operationalized as a rigid belief that all students should demonstrate 100% time-on-task 100% of the time, then high expectations become unrealistic and developmentally inappropriate expectations. The notion that young children should be able to effectively self-regulate all of the time is not consistent with our knowledge of human development, for the reasons stated above: (1) it takes a long time to build self-regulation, and children do not master this body of skills linearly but instead go through gradual cycles of progression and regression, needing to learn and re-learn skills under new and different circumstances (Aber and Jones, 1997; Best and Miller, 2010), and (2) self-regulatory skills are very sensitive to context, such that support or lack of support (e.g., from a teacher, caregiver, or peer) or specific obstacles in the environment, including stress, anger, and anxiety can interfere with a person’s capacity to access and use the skills that they otherwise have (Noble et al., 2005, 2007; Jones et al., 2013).

In addition, schools that adopt a “no excuses” approach often have an extended school day and require students to sit still for long hours, both of which may exacerbate the strain of these expectations on children’s newly emerging skills (Balonon-Rosen, 2016). If students are held accountable to an expectation impossible for them to achieve, it can undermine their feelings of motivation, self-efficacy, and competence (Ryan and Grolnick, 1986) – feelings that are necessary for working through difficult academic, personal, and social situations and for developing the kind of persistence and focus that is needed for “good” classroom behavior as well as success in college, the workplace, and beyond (Golann, 2015; West et al., 2016). This can lead to increased frustration and anxiety for children, even children who are struggling in typical, developmentally appropriate ways. As Goodman (2013) points out,

…sanctions in these CMOs [Charter Management Organizations] are not limited to what would commonly be considered misbehavior – disrupting class by interrupting a lesson; threatening, intimidating, bullying, or fighting others; failing to produce the required assignment. They are employed for behaviors that, despite appearing innocent in themselves, are forbidden so as to foreclose the *possibility* of misbehavior (p. 92).

In fact, many of these behaviors, such as talking quietly to a peer at one’s desk or in the hallway, staring into space rather than actively tracking the speaker at all times, moving one’s hands about rather than keeping them clasped on a desk, etc. are considered developmentally appropriate for school-aged children. Preventing children from natural situations that might present the possibility of misbehavior also prevents the possibility that they learn strategies to regulate themselves in these situations so as to, ultimately, prevent the misbehavior on their own. As Dishon and Goodman (2017) argue, opportunities to practice autonomous decision-making are a central mechanism for the development and internalization of these skills.

Young children arrive at school with a wide range of skills and dispositions, particularly in the domain of self-regulation. Approaches to discipline that are rigid and prescriptive without flexibility for individual differences are bound to contribute to over-punishment, right at a time of major transition – a time when children require support from adult caregivers in order to build key skills and to adapt to the complex demands of a new social and instructional environment (Rimm-Kaufman et al., 2000). Unfortunately, rather than teaching skills, some “no excuses” schools resort to shaming as a way to control behavior. Children are publicly reprimanded, made to be silent all day, or required to wear a special shirt that indicates that they have misbehaved (Goodman, 2013). Yet, research tells us that excessive anxiety and other negative feelings during this time can disrupt important brain development and learning and can interfere with key developmental tasks of the childhood years: developing self-regulatory skills, adjusting to the school environment, and having positive feelings about oneself and one’s ability to learn such as a sense of competence, mastery, and academic self-efficacy (Aber and Jones, 1997; Denham, 2006; Brion-Meisels and Jones, 2012; Center on the Developing Child at Harvard University, 2015; Bailey and Jones, 2019). As described by Reeve et al. (2012),

Greater autonomy and more positive functioning result only when the context nurtures and supports that tendency, whereas all too often these inherent positive resources are derailed or blocked by excessive controls that thwart autonomy, excessive demands that thwart feelings of competence, or an absence of warmth and care that thwarts relatedness to those who teach (p. 228).

Many “no excuses” schools emphasize the use of rigid structures and continuous teacher feedback about behavior, presumably intended to provide a sense of predictability, consistency, and support (Balonon-Rosen, 2016). Adult consistency and support are important—especially for young children (e.g., Jones and Zigler, 2002); but, if adult support is enacted as a rigid and overly prescriptive attempt to manage children’s everyday behaviors, then adult support becomes adult over-control. The development of self-regulation and social-emotional skills requires many opportunities for children to practice – sometimes with scaffolded, adult-led intervention and interaction, and other times without the presence of an adult, in order to learn how to take on the role for oneself (Sameroff, 2010). This is the very essence of “self-” regulation: taking responsibility for oneself to remember and enact strategies and problem-solve or persist through a difficult feeling or situation (Calkins, 2007; Blair and Diamond, 2008; McClelland and Cameron, 2012). Students cannot do this if they are given no opportunities to sit, walk, speak, and interact with materials, space, and each other freely. While research on self-regulation has encouraged the use of visual reminders such as posters and non-verbal cues and physical mediators such as tools to help children “cool down” when angry, take turns, share, and deal with conflicts when they arise, research does not suggest that adults should manage these interactions entirely nor manage the classroom environment in ways that prevent children from encountering and dealing with issues themselves.

To the contrary, recent research suggests that less-structured time is linked to the development of key self-regulation-related skills (Barker et al., 2014), presumably because it affords children the opportunity to exercise autonomy, rally their own motivation to support focus and persistence, and independently practice managing their time and attention – all cornerstones of adult self-regulation and effective self-management. In their 2014 study, Barker et al. found that children ages 6–7 years who spent more daily/weekly time in highly structured activities performed *less* well on measures of self-directed executive function and goal-directed behavior. Similarly and somewhat paradoxically, a study by the Harvard Center for Education Policy Research found that children in schools characterized by a highly prescriptive approach to behavior and discipline were more likely to report *lower* scores in grit, conscientiousness, and self-control than their peers in schools with a less prescriptive approach to behavior (West et al., 2016). This is consistent with studies that have found no impact of “no excuses” schools on student non-cognitive skills (e.g., Tuttle et al., 2015). When the implicit and explicit messages students receive from school staff equate self-regulation and leadership with silence, conformity, and compliance, students’ social and emotional growth can be undermined (Soutter, 2019).

### Potential Unintended Consequences

Though less well understood, there may be additional consequences of “no excuses” discipline practices that extend beyond the school day. Sending children home with 8-h-worth of frustration, anxiety, and pent-up energy risks further taxing a family system that can be, in many situations, already under extreme and chronic stress. Furthermore, there is evidence that “no excuses” behavior policies do not allow students to develop the self-management and self-regulation skills needed for success in a more autonomous environment, such as when they move up to a different school or other setting. In particular, students who may have been academically successful in the “no excuses” environment can struggle with the transition from high school to college, where there is less adult supervision and control, and some drop out before completing their degree. KIPP, one of the best known “no-excuses” charter organizations, found that while KIPP graduates were completing college at higher rates than the national average, college persistence rates were lower than expected (44%) given students’ achievement on standardized assessments and KIPP’s college admission rates overall (KIPP Foundation, 2011; Shapiro, 2019). Sondel (2016) points out, “Perhaps this is because the “no excuses” schools prepare students to accumulate predetermined content rather than exercise the skills of critical thinking and communication often expected in higher education and middle-class careers” (p. 184). We argue that, in addition, perhaps students were not afforded the opportunity to build and practice key self-regulatory skills that are essential to self-directed learning and successfully navigating the college environment. Other researchers worry that “no excuses” schools impede the development of essential life skills such as intrinsic motivation, rigorous analytic reasoning, and self-respect (Lamboy and Lu, 2017). These theories have not yet been subject to systematic evaluation, but they represent important questions to address in future research. More research into unintended negative consequences is needed to understand the full impact of “no excuses” policies on student well-being, behavior, and healthy development (Lamboy and Lu, 2017).

### Rigid Disciplinary Policies Jeopardize the Relationship Between Students and Teachers

When unrealistic expectations are coupled with overtly public and harsh, punitive consequences for not being successful – eliciting recurring feelings of shame, embarrassment or anxiety – the possibility of maintaining positive relationships or positive emotional climate is diminished, and students’ engagement in school is likely to decline over time, as is their likelihood to try harder next time (Hamre and Pianta, 2001). In a recent ethnographic study, the author spent 18 months engaged in fieldwork at a “no excuses” charter school serving primarily low-income students of color (Golann, 2015). In a series of interviews with students and staff, individuals described their experiences in a culture of high expectations coupled with a rigid, punitive disciplinary system. Students reported a lack of autonomy and few opportunities to make decisions or their own mistakes, a negative attitude toward school and teachers, feelings of stress and anxiety that overshadowed any positive learning experiences, lower motivation, and strained relationships – a feeling “at odds rather than at ease with teachers” (Golann, 2015). Golann found that teachers were disciplining students so swiftly and frequently that they would often make mistakes, either reprimanding the wrong student or failing to recognize when a student was helping rather than distracting another student, all the while demanding that student defer to their authority. The students felt that they were not respected, nor did they have a voice. One student shared, “…she didn’t care, like, she’ll go based on what she thinks she saw or heard and you wasn’t able to explain what you actually did or said” (Golann, 2015, p. 112). Another student explained how it was difficult to build relationships with teachers:

Cause some teachers, in class, you’re like, you’re so strict, I don’t want to talk to you… So like in class, like the teachers always seem like the bad person and the criminal in our mind. It’s not that easy to relate to the teachers or wanna talk to them at school (p. 113).

Golann contrasts this “no excuses” culture with other school environments where students are encouraged to think flexibly, creatively, and independently, where negotiation and question-asking are seen as key to learning and social mobility, and where students are given freedom and autonomy in decision-making.

A “no excuses” approach creates extremely high pressure not only for students but for teachers. Former teachers and observers describe a culture of “coordinated, institutionalized response” to children’s expressions of emotion, including in which teachers are instructed not to comfort 4- and 5-year old students when they cry, even in the case of a student whose parent was shot on the first day of school (Ben-Porath, 2013; Lamboy and Lu, 2017). Under such a tight, high-stakes accountability system, unrealistic expectations for children’s behavior may contribute to increased frustration, punitive practices, and negative communication, leading to poorer quality relationships between teachers and students, lower levels of emotional support, and heightened stress in the classroom. These factors are likely to exacerbate the problem behavior – not improve it – and lead to increased teacher burn-out and high turn-over in staff (Taylor, 2015; Torres, 2016). Many teachers find it draining to be constantly scanning for and vigilant about rigid behavior expectations (e.g., silent, sitting up straight, hands clasped, always tracking the speaker, etc.), leading to increased negative interactions with students and an overall feeling of failure (Torres, 2016). Torres (2014) found that, after controlling for workload and teacher characteristics, teachers’ perceptions of disciplinary systems were associated with teacher turnover in a sample of New York City Charter Management Organizations (CMOs). When there are high levels of teacher turn-over, we expect it also becomes difficult to establish strong relationships and trust between the school staff and families and the larger community, all of whom play an integral role in the development of the whole child.

### Reactionary Discipline Perpetuates Structural Inequality

Finally, and perhaps most importantly, no excuses policies contribute in dangerous ways to the racial and socioeconomic inequalities that pervade U.S. schooling. “No excuses” charter schools primarily operate in low-income communities and currently make up a majority of the charter school sector in many urban centers of the US (Angrist et al., 2013). As a result, many low-income children and children of color attend schools that are characterized by adult over-control and lack opportunities for young children to practice essential self-regulation and social and emotional skills through everyday interactions (Kahlenberg and Potter, 2014; Golann, 2015). On the other hand, more affluent schools often prioritize unstructured time and self-directed learning (e.g., Barker et al., 2014). These different approaches are symbolic of a problematic understanding of child development in the United States. The misbehavior of low-income children and children of color is frequently seen as an inability to self-regulate and thus requiring strict adult control, while misbehavior in wealthy or white contexts is often presented as exploration, positive risk-taking, or creativity (Green, 2018). As Golann (2015) explains, this reinforces racial and class-based divides, as low-income children of color are taught to be “worker-learners – children who monitor themselves, hold back their opinions, and defer to authority,” rather than given the opportunity to develop key social and emotional learning skills such as critical thinking, problem-solving, and creativity, that are required for success in the modern economy. Limiting essential skill-building and independent, critical thinking fails to prepare students for democratic citizenship and participation in civic life (Lack, 2009; Dishon and Goodman, 2017). In this way, schools adopting a “no excuses” approach to behavior, who serve primarily low-income students and students of color, may unwittingly reinforce and exacerbate existing inequalities in the broader society.

In addition, a “no excuses” approach may lead to more suspensions and expulsions, both of which are linked to poorer academic achievement (Morris and Perry, 2016) and to negative developmental outcomes later in life (Council on School Health, 2013; Meek, 2014; Skiba et al., 2014). Recent reports document that race-based discipline disparities have increased dramatically in the US over the past 40 years (Losen and Martinez, 2013; Rumberger and Losen, 2016), which may be partially attributed to increasing use of “no excuses” and “zero tolerance” discipline practices in schools serving primarily low-income and African American communities (Opportunities Suspended: The Devastating Consequences of Zero Tolerance and School Discipline, 2000). As described above, rigid disciplinary policies that require teachers to be constantly and quickly doling out rewards and punishments inevitably lead to misinterpretations and mistakes, often driven by implicit biases (Staats, 2014; Gilliam et al., 2016; Gregory and Fergus, 2017). It is not uncommon to hear children, particularly children of color, say, “The teacher only sees me” (Green, 2018). In a review of kindergarten disciplinary referrals, former Minneapolis schools superintendent Bernadeia Johnson found that teachers described white students with behavior challenges as “gifted but can’t use his words” and excused their actions because they “had a hard day,” whereas they described black children as “destructive,” “violent,” and “cannot be managed” (Green, 2018). This reflects a trend of exclusionary discipline policies disproportionately affecting low-income children, children of color, and children with disabilities (Cortiella and Horowitz, 2014; Rumberger and Losen, 2016; Rafa, 2019).

Exclusionary discipline policies can be especially detrimental to students who have experienced trauma, in part because the act of social exclusion is often re-traumatizing (Marcus, 2014; Cole et al., 2015; Lamboy and Lu, 2017). In one study conducted with social workers from three “no excuses” charter schools in New York, Balogh (2016) reported that the social workers expressed deep concerns about how the rigid and one-size-fits-all behavioral expectations disproportionally impact children of color, students with disabilities such as ADHD, and children who have experienced trauma. One social worker stated:

It sickens me sometimes to feel like we replicate something that can bring up something very traumatic for a student of color or a student of trauma. So if you’re a student who has been disenfranchised, felt isolation, felt rejected, not necessarily felt heard for whatever outside reason, and you can come into this school and on some level we replicate that—not on a conscious level, on an unconscious level…I tend to believe because also we get students of color who racially have felt a lot of stuff, that even brings up even more stuff for them…They battle this idea of “I have no voice. Why do I have to sit in this damn room for eleven hours? Why can’t I operate this particular way?” (personal communication, November 4, 2015) (Balogh, 2016, p. 21).

Another social worker shared:

My training and practice teaches me that the way to support children with certain diagnoses is not aligned with those behavioral expectations…A lot of the children with ADHD were repeatedly spending a lot more time in the dean’s office than they were in the classrooms because they were physically unable to meet those expectations without scaffolding them to get there. And it was significantly impacting their self-esteem and I was repeatedly hearing in sessions—and ADHD is just one example—that they were bad and couldn’t do good (personal communication, December 9, 2015) (Balogh, 2016, p. 21–22).

Suspensions and expulsions may push the problem out of the classroom or school in the short-term, but they lead to negative long-term outcomes for individual children as well as for school systems and the communities they serve. For example, extensive research documents the link between suspensions and the probability of dropping out of school (Suh and Suh, 2007; Skiba et al., 2014). Students who drop out of high school earn an average of $375,000 less than high school graduates and an average of $1 million less than college graduates over the course of their lifetime. In addition, it has been found that they are eight times more likely to be incarcerated than students who complete high school (Christle et al., 2005; Center for Labor Market Studies, 2009; Skiba et al., 2014). One study estimated the U.S. economic impact of suspensions to be $11 billion in lost tax revenues and more than $35 billion in costs to society (Rumberger and Losen, 2016). In contrast, school and community investments in proactive approaches to behavior management such as social and emotional learning have shown an economic return-on-investment of 11 to 1 (Belfield et al., 2015).

## What does the Research Tell us we Should do Instead?

### Explicitly Teach Self-Regulation Skills Through Activities and Routines

There is a growing body of research describing the characteristics of environments that promote self-regulation and other social emotional skills (e.g., Boyd et al., 2005; Diamond and Lee, 2011; Durlak et al., 2011). Schools that effectively support the development of self-regulation typically do so in three primary ways (Jones and Bouffard, 2012; Jones et al., 2019). First, they provide children with direct learning and experience with specific skills, such as **cognitive regulation** (e.g., focus, inhibitory control, working memory, flexibility, and planning), **emotional competencies** (e.g., recognizing and communicating feelings, demonstrating empathy), and **prosocial behaviors** (e.g., understanding others, cooperating, turn taking, helping others, and conflict resolution) (Aspen Institute National Commission on Social, Emotional, and Academic Development, 2019). Second, they draw on a set of daily routines and weekly activities that allow children multiple opportunities to practice—to try out using new skills and receive feedback—regularly (e.g., exercising self-control in settings where children have real choice and independence, or practicing emotion management by voicing one’s feelings and negotiating between different opinions or perspectives) (Bailey and Jones, 2015; Dusenbury et al., 2015). Third, they are developmentally aligned to children’s emerging skill areas and grow progressively more complex and require less adult support over time (Bailey et al., 2019; Jones et al., 2019).

Programs that are most effective typically meet the SAFE criteria: (S) **sequenced** activities to develop skills, (A) **actively** engage students in learning and practicing skills, (F) **focused** time on social and emotional skill development, and (E) **explicitly** define and target specific social and emotional skills (Jones et al., 2017, 2019). For example, the SECURe program was designed as a universal, school-based intervention that combines executive function, self-regulation, and social and emotional skills with a high-quality language and literacy curriculum for children in grades PreK-3, using a set of daily classroom and school-wide structures and routines and weekly classroom lessons focused on social and emotional skills necessary for learning (e.g., active listening, paying attention, understanding feelings, and resolving conflicts) (Jones et al., 2014; Jones and Bailey, 2014). In this program, students are given daily opportunities to practice focus, impulse control, and working memory through a set of games that can be played in classrooms or in other settings in schools that strengthen their use of these skills; the games are designed to be fun, playful, and motivating. Students also engage in “feelings circles” and weekly “class council” meetings where they discuss and solve problems together; and students learn effective communication and conflict resolution strategies *via* role play, videos, and open-ended discussion of scenarios (Jones et al., 2016). Results from a small randomized evaluation of SECURe showed positive effects on children’s attention/impulsivity as well as positive and statistically significant effects of the program on growth in both reading and math achievement over the course of the school year (Jones et al., in review).

Even amidst the pressure to spend increasing amounts of time on test prep, these strategies and routines are valued as an everyday part of the school’s work because teachers recognize that the skills underlying self-regulation and social-emotional development are essential to children’s success in school (Durlak et al., 2011) and to their overall health and well-being (Moffitt et al., 2011; Jones et al., 2015). Actively building these skills enables children to get along with others, persist with challenging tasks, and focus and remember key information – supporting academic learning as well as positive behavior, both within school and beyond. In contrast, when schools adopt zero tolerance policies that send children out of the room for minor distractions, disruptions, and peer conflicts, they prevent teachers from engaging *with* students through typical, developmentally appropriate challenges, directly undermining students’ opportunities to learn and practice these critical skills.

### Create a Warm and Positive Environment Characterized by Responsive Relationships and Adult Modeling

Schools that effectively support the development of self-regulation expect adults to model these strategies in real time and scaffold their use for students. Children in these schools watch adults use self-regulation strategies in context, when difficult situations arise; and, when these children struggle with attention or behavior, they are not reprimanded but instead coached to practice using the same strategies (e.g., Lewis, 2015). This requires schools to focus not only on student skill-building, but also on building adult capacity through professional development, coaching, and supportive policies and practices that promote teachers’ own self-regulation and social-emotional development (Schonert-Reichl, 2017; Bailey and Jones, 2019). Professional development in social emotional learning and positive behavior management is shown to improve classroom climate and instructional practices, as well as individual child outcomes (e.g., Jones et al., 2013). Effective professional development provides adults with the training and supports to build positive connections with students, de-escalate conflicts, implement constructive interventions, and creates supportive conditions for learning, such as high-quality relationships and teacher modeling of key skills (Morgan et al., 2014).

In addition to explicit modeling, adult mindsets with regard to discipline directly impact the quality of adult-child relationships as well as rates of suspension (Okonofua et al., 2016). Integral to this work with adults is a focus on equity and culturally sustaining pedagogy (Paris, 2012), in which educators are able to reflect on the “disciplinary moment” with students in order to counteract implicit biases that contribute to disproportionate rates of suspension for children of color (Lustick, 2017). Teachers are then given time, tools, autonomy, and support to build warm and trusting relationships founded on high expectations for all students and to respond more equitably in practice (Ladson-Billings, 1995; Hammond, 2015). Okonofua et al. (2016) found that an intervention that encouraged an empathic mindset among teachers by reminding them of the importance of positive student-teacher relationships for students’ growth and non-pejorative reasons why children misbehave cut suspension rates in half from 9.6 to 4.8%. Teachers were empowered to take ownership of the intervention message, to make connections to their own practice, and to share the message with future teachers through written reflection. Finally, teachers’ empathic responses directly impacted the quality of their relationships with students; students who were previously suspended felt more respected by their teachers who had taken part in the intervention than those who had not (Okonofua et al., 2016).

Children learn best through learning that is interactive and relational, rather than unidirectional (National Scientific Council on the Developing Child, 2004). Successful schools provide an emotionally supportive climate characterized by positive interactions and relationships that are responsive to individual needs, and warm, nurturing, and empathic relationships, such that students feels safe to try, fail, and work together to overcome obstacles and learn from their mistakes (Brown et al., 2010; Li and Julian, 2012; Thapa et al., 2012).

### Shift Policies and Practices Toward Positive and Proactive Behavioral Supports

Increasingly, schools, districts, and states are recognizing the negative and inequitable consequences of “zero tolerance” disciplinary policies, particularly for marginalized students. Over the past 5–10 years, a number of bills have passed that limit the use of exclusionary discipline practices such as suspensions and expulsions and promote the use of non-punitive, alternative practices that rely on positive and proactive behavioral interventions (Gregory and Fergus, 2017; Rafa, 2019). In 2013, the American Academy of Pediatrics published a policy statement outlining the severe consequences of “zero tolerance” discipline practices on the developing child and encouraged pediatricians to take stronger steps to discourage suspensions and expulsions (Council on School Health, 2013). The following year, the Council of State Governments issued a consensus report on school discipline which also discouraged use of exclusionary practices (Morgan et al., 2014). As of 2019, 16 states and Washington, D.C. have laws that limit the use of suspensions and expulsions, largely in the early grades. In addition, the Every Student Succeeds Act (ESSA) now requires all states to collect data on numbers of in- and out-of-school suspensions, in addition to at least one School Quality and Student Success Indicator (SQSS), which are included on state school report cards (Rafa, 2019). Funding is also available through ESSA to support initiatives to promote a positive school climate, which may include social and emotional learning and positive behavioral support initiatives (Grant et al., 2017).

Alternative discipline strategies such as Schoolwide Positive Behavioral Interventions and Supports (SW-PBIS) and Restorative Justice practices have been shown to be effective not only in improving behaviors and decreasing the amount of time that students are out of the classroom, but also in increasing academic achievement and student engagement (González, 2014; Augustine et al., 2018; Rafa, 2019). Schoolwide PBIS encourages adults to clearly define expectations and, importantly, to focus on noticing and reinforcing positive behavior (SWPBIS Implementation Blueprint, 2010). PBIS is grounded in a body of research showing that adverse consequences for problem behavior tend to be least effective for students with the most severe behavior problems and do not lead to improved behavior (e.g., Shores et al., 1993; Tolan and Guerra, 1994; Walker et al., 1996). PBIS has a proven, positive impact on *all* students, including students with learning disabilities, who often face challenges with self-regulation skills and who experience disproportionately high rates of suspension and expulsion (Cortiella and Horowitz, 2014).

Restorative Justice practices aim to repair harm, restore relationships, and build community; they encourage collaborative problem-solving rather than the doling out of consequences and give voice to both the person harmed and the person who caused the harm (Wachtel et al., 2009; Gregory and Fergus, 2017). In practice, individuals may sit in a circle facing each other, reflect on a prompt or question, and take turns sharing perspectives. The goal is to work toward a solution and re-entry to the classroom. Emerging research suggests restorative practices are an effective way to increase positive behavior and reduce race-based discipline disparities, while keeping students in school (McCluskey et al., 2008). For example, the Denver Public Schools saw a decrease in suspension rates from 10.58 to 5.63% between 2006 and 2013 after adopting a restorative justice model across the district schools as an alternative to zero tolerance and exclusionary discipline policies. During this time, suspension rates for African American students fell the most in absolute terms (7.2%), and the suspension gap between White students and African American students decreased by almost 4% to approximately an 8-percentage point gap (González, 2014). At the same time, the district saw a steady increase in academic achievement, as measured by the percentage of students scoring proficient or above on statewide standardized assessments, average ACT scores, and on-time graduation rates (González, 2014). Similarly, Ascend charter network eliminated its “no excuses” disciplinary policy in which students’ behavior was publicly displayed through clips on a color chart (student clips start the day on green for good behavior, but are moved to yellow, and then red for minor misbehaviors), and saw suspensions fall by 40% and test scores in English and math rise by almost 35 percentage points (Shapiro, 2019). Ascend’s chief executive officer shared, “The conversation in the bustle of the kids leaving was, ‘What color were you on today?’… Not what did you learn, or what excited you, or what did you discover? We thought this was just tremendously sad” (Shapiro, 2019).

Finally, in addition to proactive and restorative approaches to behavior management, schools can adopt trauma-informed practices that focus on relationships and allow teachers and administrators to better understand and consider a child’s background and experiences when creating systems, structures, and responses related to discipline (Cole et al., 2015). Many schools integrate these practices within a multi-tiered system of support model, in which universal school-wide implementation of PBIS is complemented by restorative practices and trauma-sensitive supports that reflect an individualized plan of care developed by a collaborative team (Gregory and Fergus, 2017).

## Conclusion

Charter schools are expanding rapidly in the U.S. public education sector, with more than 2.5 million students currently attending charter schools (National Alliance for Public Charter Schools, 2014; Snyder et al., 2016). A majority of the charter schools in many urban, low-income communities have adopted a “no excuses” approach to classroom management practices that are characterized by strict adult-control of children’s behavior (Angrist et al., 2013), though this trend may be shifting based on recent findings, as described above. “No excuses” practices run counter to what we know from the science of human development about children’s regulatory skills, which suggests that the early years of school are a central context for learning self-regulation through explicit teaching and practice and through modeling from supportive caregivers and peers. More research is needed to isolate and test the short- and long-term impacts of “no excuses” disciplinary policies on children’s social and emotional development. We argue that unreasonably high expectations that do not align with children’s developmental skill progression can lead to frustration and anxiety, undermining children’s feelings of self-efficacy and motivation. These policies also jeopardize the relationship between students and teachers when strict compliance is prioritized over building relationships with and being responsive to individual students’ needs. Finally, we believe that “no excuses” disciplinary policies, which largely impact low-income students and students of color perpetuate race, class, and ability-based inequities through policies that disproportionately exclude students who are most vulnerable and who most benefit from explicit teaching and learning of social and emotional skills. High-quality social and emotional learning programming and responsive and restorative practices are not only evidence-based approaches to managing behavior in classrooms, they are also tied to long-term positive outcomes (Durlak et al., 2011; González, 2014; Belfield et al., 2015; Jones et al., 2015; Mahoney et al., 2018). School policies and practices should build upon the developmental science literature showing the importance of social and emotional skills to children’s academic and behavioral outcomes by explicitly teaching skills and providing opportunities to practice, focusing on relationships and adult competencies, and implementing positive and trauma-informed behavioral supports.

## Author Contributions

SJ, RB, and GB-M contributed conception and design of the paper. RB and GB-M wrote the first draft of the manuscript. EM conducted additional research and wrote sections of the manuscript. All authors contributed to manuscript revision, read, and approved the submitted version.

### Conflict of Interest Statement

The authors declare that the research was conducted in the absence of any commercial or financial relationships that could be construed as a potential conflict of interest.

## Footnotes

^1^Self-regulation is our umbrella term that represents the ability to manage thinking, attention, feelings and behavior. It overlaps with many other terms including grit, willpower, self-control, and executive function, among others. We use the term “self-regulation” throughout this paper to refer to this broad area of children’s development. See Bailey and Jones, (2019) for more on self-regulation and executive function/related skills.
